# The Impact of Polycomb Group Proteins on 3D Chromatin Structure and Environmental Stresses in Plants

**DOI:** 10.3390/plants14071038

**Published:** 2025-03-27

**Authors:** Yali Liu, Suxin Xiao, Minqi Yang, Guangqin Guo, Yue Zhou

**Affiliations:** 1 Institute of Cell Biology and MOE Key Laboratory of Cell Activities and Stress Adaptations, School of Life Sciences, Lanzhou University, Lanzhou 730000, China; liuyl20@lzu.edu.cn; 2 State Key Laboratory of Gene Function and Modulation Research, School of Advanced Agricultural Sciences, Peking-Tsinghua Center for Life Sciences, Peking University, Beijing 100871, China; 2201112380@stu.pku.edu.cn (S.X.); yangmq@stu.pku.edu.cn (M.Y.)

**Keywords:** PRC1, PRC2, H3K27me3, H2Aub, 3D chromatin structure, environmental stresses

## Abstract

The two multi-subunit complexes, Polycomb Repressive Complex 1 and 2 (PRC1/2), act synergistically during development to maintain the gene silencing state among different species. In contrast with mammals and *Drosophila melanogaster*, the enzyme activities and components of the PRC1 complex in plants are not fully conserved. In addition, the mutual recruitment of PRC1 and PRC2 in plants differs from that observed in mammals and *Drosophila*. Polycomb Group (PcG) proteins and their catalytic activity play an indispensable role in transcriptional regulation, developmental processes, and the maintenance of cellular identity. In plants, PRC1 and PRC2 deposit H2Aub and H3K27me3, respectively, and also play an important role in influencing three-dimensional (3D) chromatin structure. With the development of high-throughput sequencing techniques and computational biology, remarkable progress has been made in the field of plant 3D chromatin structure, and PcG has been found to be involved in the epigenetic regulation of gene expression by mediating the formation of 3D chromatin structures. At the same time, some genetic evidence indicates that PcG enables plants to better adapt to and resist a wide range of stresses by dynamically regulating gene expression. In the following review, we focus on the recruitment relationship between PRC1 and PRC2, the crucial role of PcG enzyme activity, the effect of PcG on 3D chromatin structure, and the vital role of PcG in environmental stress in plants.

## 1. Introduction

Animals and plants comprise a multitude of different cell types and, with a few exceptions, all of these cell types within an individual contain the same DNA encoding genetic information. The uniqueness of particular cell types is essential to maintaining specific developmental processes during growth and cell division. PcG proteins play a crucial role in stabilizing and inheriting transcriptional silencing [1]. Polycomb (Pc) was the first polycomb protein identified in *Drosophila*. It was named as such following the observation that *Pc* mutants develop extra sex combs on their second and third legs. The *Pc* mutant displays embryonic lethality, characterized by all cuticular segments resembling the final abdominal segment. These phenotypes arise due to the ectopic expression of *Homeobox* (*HOX*) genes [2]. PcG proteins are divided into two major groups based on their biochemical characteristics: PRC1 and PRC2. PRC1 and PRC2 are evolutionarily conserved across multi-cellular eukaryotes [3,4,5,6]. PRC1 is an H2A E3 ubiquitin ligase complex, and PRC2 is an H3 lysine 27 methyltransferase complex.

The PRC1 subunits in *Drosophila* are Sex combs extra (Sce, alternatively named dRing1), Posterior sex combs (Psc), Suppressor of zeste 2 (Su(z)2), Pc, Polyhomeotic (Ph), Sex comb on midleg (Scm), RING1 and Yin Yang1 (YY1) Binding Protein (dRybp), and H3K36 lysine (K)-specific histone demethylase 2B (dKdm2B). The above-mentioned PRC1 proteins in *Drosophila* contain homologous proteins in mammals (Table 1) [7,8,9]. Mammals have six distinct PRC1 complexes (PRC1.1 to PRC1.6), and each of these complexes is distinguished by a specific type of Polycomb Group Ring Finger Protein (PCGF) subunit (PCGF1 to PCGF6). Additionally, each PRC1 complex, from PRC1.1 to PRC1.6, includes either RING1A or RING1B. In contrast with mammals and *Drosophila*, no homologous proteins of Pc and Ph have been identified in plant PRC1 complexes. However, the homologs of Psc and Sce in *Drosophila*, B lymphoma Mo-MLV Insertion region 1 homologs (BMI1A/B/C) and RING1A/B have been found in plants. Additionally, plants possess unique PcG proteins, such as Like-Heterochromatin Protein 1 (LHP1) and Embryonic Flower 1 (EMF1) [7]. The *Drosophila* PRC1 core components (Psc, Sce, and Pc) can compress nucleosomes, with the Psc C-terminus primarily acting as the compressor [10]. The chromodomain of Pc binds H3K27me3 [11,12]. In *Drosophila* and mammals, Sce and RING1A/B exhibit E3 ubiquitin ligase activity, and the BMI1 protein enhances the enzymatic activity of RING1 [13,14,15,16,17,18]. In contrast, in plants, both RING1A/B and BMI1A/B/C exhibit E3 ubiquitin ligase activity [19,20,21,22,23]. LHP1 is the structural homolog of Heterochromatin Protein 1 (HP1) in mammals, but it has different functions. Both LHP1 and HP1 contain chromo and chromo shadow domains; however, the chromodomain of HP1 binds to H3K9me3, whereas the chromodomain of LHP1 binds to H3K27me3. Additionally, the chromo shadow domain of HP1 or LHP1 is essential for the formation of homodimers [24,25,26,27]. EMF1 acts as a repressor and is involved in chromatin compression [28,29].

Furthermore, the core subunits of PRC2 in *Drosophila* are Enhancer of zeste [E(z)], Suppressor of zeste 12 [Su(z)12], Extra sex combs (Esc), and Nucleosome remodeling factor 55 kDa (Nurf55). E(z) is the subunit that catalyzes H3K27 methylation [30]. In mammals and *Drosophila*, PRC2 is divided into two classes, PRC2.1 and PRC2.2; however, there exists no such detailed PRC2 classification in plants [9,31,32]. The corresponding PRC2 core subunits in *Arabidopsis thaliana* are Curly Leaf/Swinger/Medea (CLF/SWN/MEA), Embryonic Flower 2/Vernalization 2/Fertilization Independent Seed 2 (EMF2/VRN2/FIS2), Fertilization Independent Endosperm (FIE), and Multiple Suppressor of Ira 1 (MSI1) [7]. In *Arabidopsis*, there are three types of PRC2 complexes: FIS-PRC2, EMF-PRC2, and VRN-PRC2. All three complexes contain FIE and MSI1 subunits, with MEA and CLF/SWN functioning during gametophyte and sporophyte development, respectively [33]. Most PcG proteins are recruited to chromatin by other protein factors, and only a few PcG proteins are known to bind chromatin DNA directly. For example, *Drosophila* PcG proteins Pleiohomeotic (Pho) and Pleiohomeotic-like (Phol), in addition to the mammalian PcG proteins YY1/2, can directly bind polycomb response elements (PREs) [34,35]. In *Arabidopsis*, Telomere Repeat Binding factors (TRBs) and Enhancers of LHP1 (EOL1) are capable of recruiting PRC2; additionally, VP1/ABI3-LIKE (VAL) has the ability to recruit both PRC1 and PRC2 [36,37,38,39]. Other factors are also known to recruit PRC1 or PRC2 [8].

In the following review, we examine emerging studies, particularly those elucidating the PcG functions that shape 3D chromatin structure and highlighting their significance in mediating plant resilience under abiotic (heat, drought, cold, and salinity) and biotic (pathogen) stress conditions. The objective of this literature review is to synthesize recent advancements in understanding how PcG proteins in plants influence 3D chromatin structure. These mechanisms may play critical roles in regulating stress-responsive genes and enabling adaptive responses to environmental challenges such as heat, drought, cold, high salinity, and pathogen attacks.

## 2. Methods

The literature search methodology was systematically conducted through multiple complementary approaches. Primary information sources included the following: (1) Core biological databases (PubMed and TAIR) (2) Authoritative textbooks: Epigenetics (2nd Edition) and Biochemistry and Molecular Biology of Plants (2nd Edition); (3) Computational literature mapping tools (Connected Papers and Paper Digest); (4) Seminal publications from leading plant epigenetics research groups.

## 3. The Relationship Between PRC1 and PRC2

In mammals, depending on their ability to be recruited by H3K27me3, PRC1 complexes are subdivided into canonical Polycomb Repressive Complex 1 (cPRC1) and variant Polycomb Repressive Complex 1 (vPRC1). cPRC1 contains one of the Chromobox (CBX2/4/6/7/8), which binds to H3K27me3. In contrast, vPRC1 includes subunits such as RYBP or YY1-Associated Factor 2 (YAF2), which binds H2Aub. Additionally, vPRC1 can be recruited directly to chromatin by other factors, such as KDM2B [8,31,32]. RING1 and PCGF proteins can form heterodimers or homodimers; however, a full understanding of homodimers’ biological function remains elusive [13,40,41,42,43].

cPRC1 is not currently found in plants because no plant homologs of Pc/CBXs, Ph/PHCs, or SCM proteins exist. Based on research findings, plants appear to contain only vPRC1s (Table 1) [8]. It is not clear how RING1 and BMI1 proteins function combinatorially in plants—whether through homodimerization, heterodimerization, or other mechanisms in plants. In *Arabidopsis*, researchers found that RING1A interacts with itself through yeast two-hybridization and that RING1A/B interacts with BMI1A/B/C, respectively. However, the true situation in vivo remains unclear [44,45]. In the *Atring1a/b* mutant, the expression of the three *BMI1A/B/C* genes was upregulated. Conversely, in the *Atbmi1a/b* mutant, the expression of the two *RING1A/B* genes was upregulated [44].

In mammals, vPRC1 is initially recruited to chromatin to deposit H2Aub. The RYBP and YAF2 subunits within vPRC1 serve as binding proteins of H2Aub [46,47,48]. In PRC2.2, the auxiliary subunit Jumonji and AT-rich Interaction Domain containing 2 (JARID2) binds H2Aub, which facilitates the recruitment of PRC2.2 to chromatin and subsequent deposits H3K27me3. In contrast, PRC2.1 is recruited to deposit H3K27me3 through one of three mechanisms: interaction with one of the polycomb-like (PCL) proteins: PCL1, PCL2, or PCL3; association with EPOP; or binding to the PRC2-associated LCOR isoform (PALI1/2). The H3K27me3 deposited by both PRC2.1 and PRC2.2 can recruit cPRC1, and the CBX subunit of cPRC1 can specifically bind to H3K27me3 [31,32,49].

**Figure 1:**
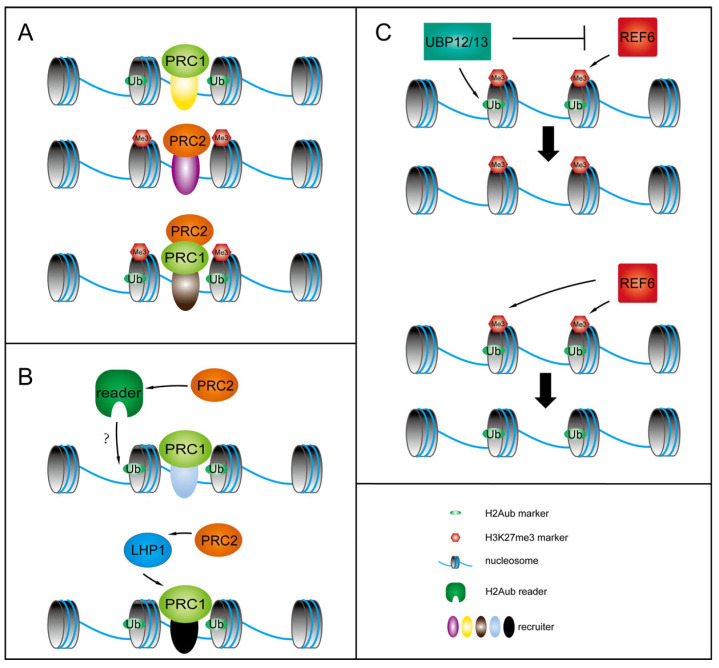
Schematic illustration of PRC1 recruiting PRC2 in plants: (**A**) model of the distribution of PRC1 and PRC2 in *Arabidopsis*; (**B**) possible ways in which PRC1 recruits PRC2 via the H2Aub reader or LHP1; the question mark indicates that the reader protein for H2Aub has not yet been identified in plants. (**C**) dynamic histone modification changes and gene activity of H2Aub- and H3K27me3-marked genes. Removing H2Aub alone does not activate the gene; instead, it remains in a repressed state. In contrast, removing H3K27me3 leads to gene activation, transitioning the gene from a repressed to an active state.

In *Arabidopsis*, it is hypothesized that PRC1 recruits PRC2; however, both PRC1 and PRC2 can also be recruited to chromatin individually and independently (Figure 1A) [50]. The exact mechanism by which PRC1 recruits PRC2, whether PRC1 recruits PRC2 through H2Aub reader proteins or protein interactions, remains undetermined (Figure 1A,B). In mammals, H2Aub binding proteins, such as RYBP, JARID2, Remodeling and Spacing Factor 1 (RSF1), and Zuotin-Related Factor 1 (ZRF1) have been identified. However, unlike mammals, no H2Aub binding proteins have yet to be discovered in plants. In mammals, ZRF1 is responsible for removing ubiquitin to enhance transcriptional activation [51]. In contrast, in the *Atzrf1a/b* mutant of *Arabidopsis*, seed development genes are upregulated, and levels of H2Aub and H3K27me3 are reduced. The Ubiquitin-binding Domain (UBD) of AtZRF1b can function to maintain the H2Aub modification level independent of PcG proteins. Consequently, the role of H2Aub readers in plants remains poorly understood [52]. Research indicates that the RING1A of PRC1 can interact with the CLF of PRC2 and that LHP1 interacts with both PRC1 and PRC2 (Figure 1A,B) [7,44,45]. Ubiquitin-specific Proteases 12 and 13 (UBP12/13) can interact with LHP1 in vivo and in vitro [53]. UBP12/13 functions as deubiquitinating enzymes specific to H2Aub; in contrast, Relative of Early Flowering 6 (REF6) serves as a demethylase targeting H3K27me3. REF6 shows a tendency to preferentially remove H3K27me3 from genes that are marked with H2Aub rather than from those that lack this modification. This ability enables REF6 to selectively activate the expression of particular genes. The activity of REF6 is positively correlated with the responsiveness of genes: genes with H2Aub are more prone to activation; in contrast, genes with H3K27me3 are more likely to be repressed. REF6 facilitates the swift response of genes with H2Aub to external stimuli by removing H3K27me3, thereby transitioning these genes from a state of stable repression to activation. Conversely, UBP12/13 counteracts REF6’s action by removing H2Aub, thus preserving PRC2-mediated gene repression (Figure 1C) [53,54,55].

## 4. The Critical Role of H3K27me3 and H2Aub

Mutants in the catalytic core subunits of PRC1 and PRC2 lead to severe developmental phenotypes. With regard to PRC1 catalytic subunit mutant phenotypes, in mice, *RING1A/B*-deficient embryos are stunted at the two-cell stage [56]. In *Arabidopsis*, mutation of *RING1A/B* leads to ectopic meristem formation and defects in floral organ development, including abnormal leaf primordium formation, alterations in carpel shape and number, ovule development defects, and obstruction of embryo sac formation. RING1A interacts with CLF and LHP1, suggesting a synergistic effect between the PRC1 and PRC2 complexes in inhibiting the expression of class I *KNOTTED-like homeobox* (*KNOX*) genes in the shoot apical meristem. RING1A/B, by binding to the chromatin of *Argonaute* family genes (*AGO*) and key transcription factors such as WRKY23 and REM34/35, promotes H2Aub deposition, thereby inhibiting the expression of these genes and regulating the development of the female gamete [45,57,58]. Mutations in *BMI1A/B/C* led to a more severe phenotype. Specifically, the leaves and roots develop embryo-like structures, including twisted leaves and embryonic tissues. BMI1s mediate the H2Aub via the E3 ubiquitin ligase activity of the RING finger domains. H2Aub modification is a crucial marker of gene silencing. When BMI1 proteins become unfunctional, the H2Aub marker cannot be formed or maintained properly. Consequently, some genes that should be silenced at specific developmental stages, such as those genes related to embryonic traits, are inappropriately reactivated during vegetative growth [19,20,22,38,44]. These abnormal phenotypes highlight the essential role of H2Aub in regulating *Arabidopsis* development.

In mice, *EZH2* encodes *the* PRC2 catalytic subunit and is predominantly expressed in proliferating cells, such as embryonic stem cells, whereas *EZH1* is mainly expressed in later developmental stages, including differentiated cells. EZH2-KO mice exhibited abnormal embryonic morphology and died approximately 8.5 days post-fertilization (E8.5) [59,60]. In *Arabidopsis*, MEA is essential in maternal expression to limit embryonic cell proliferation [61,62]. The *mea* mutant embryo overproliferates and dies during seed drying [63]. Although the endosperm can develop autonomously even in the absence of fertilization, this development remains incomplete [64]. MEA is likely to regulate the expression of its target genes through histone modification, specifically H3K27me3. In the *mea* mutant, the loss of MEA function may result in the deregulated expression of its target genes (such as *PHERES1*), leading to excessive cell proliferation [65]. Additionally, the loss of the maternal *MEA* allele disrupts the normal silencing of paternal alleles, thereby affecting the regular development of the seeds [61,66]. The *Arabidopsis* mutant *clf/swn* phenotype is characterized by a loss of differentiation ability, which prevents the formation of normal tissues or organs. Specifically, the *clf/swn* mutant is unable to make the transition from embryonic to vegetative growth and retains embryonic characteristics [29,67]. Mutation in *CLF* and *SWN* results in the inability to catalyze H3K27me3 modification. This loss of H3K27me3 modification leads to abnormal expression of PRC2 complex target genes, which subsequently affects plant growth and development [67,68]. H3K27me3 histone modification, catalyzed by PRC2 complex subunits such as EZH1/2 in mice and MEA/CLF/SWN in *Arabidopsis*, is crucial for regular embryonic development and differentiation.

During embryonic development in *Drosophila*, PRC1 can partially repress target genes even in the absence of H2Aub, likely through non-catalytic mechanisms (e.g., chromatin compaction). However, the SceI48A mutant (SceI48Am-z-) dies at the end of embryogenesis, suggesting that H2Aub activity is critical for silencing specific genes [15]. The site RING1BI53 is conserved in mammals, corresponding to *Drosophila* SceI48, and mutation at this site disrupts the interaction between E2 and E3. The cortical neural progenitor cells of mouse embryos infected with RING1BI53A showed a band upon long exposure to H2Aub immunoblotting compared to RING1BI53A/D56K. Therefore, the mutation of RING1BI53A or SceI48A is likely to retain residual enzyme activity [13,69]. The importance of the enzymatic activity of PRC1 in *Drosophila* requires further investigation. Studies involving the use of RING1BI53S and RING1BI53A/D56K mammalian embryonic stem cells have demonstrated the essential role of H2Aub in gene repression [31,32]. During neuronal fate restriction in the mouse neocortex, the PRC1 complex employs two distinct mechanisms to repress gene expression: one is dependent on ubiquitination, and the other is independent of ubiquitination [69]. During mammalian DNA damage repair, the RING-BMI complex is recruited and subsequently ubiquitinates H2AX [70,71,72]. In addition to repressing gene expression, H2Aub in mammals appears to be associated with gene activation. Genes with bivalent chromatin modifications—characterized by both H2Aub and H3K4me3—are in a poised state, ready for activation [73,74,75].

Unlike in mammals, *Arabidopsis* RING1A/B and BMI1A/B/C proteins exhibit E3 ubiquitin ligase activity [19,20,21,22,23]. In *Marchantia polymorpha*, H2Aub-deficient mutants (H2AK115R/K116R and H2AK119R) and an *Mpbmi1/1l* mutant were constructed. These mutants that prevent H2Aub deposition exhibited severe growth retardation and morphological defects. H2Aub directly promotes the deposition of H3K27me3 in *Marchantia polymorpha*, suggesting that PRC1 plays a critical role in the polycomb repression system [76]. In addition to ubiquitinating H2A, PRC1 can ubiquitinate other variants of H2A. The transcriptional regulation of H2A.Z in *Arabidopsis* is repressed by AtBMI1, and mono-ubiquitinated H2A.Z exerts a repressive effect on transcription [77]. Studies conducted thus far have only shown that PRC1 in plants can ubiquitinate H2A and H2A.Z, and it remains unclear whether other H2A variants can be ubiquitinated by PRC1 and what their functions might be [78]. The relationship between H2Aub and H3K4me3 in plants remains unclear. In *Arabidopsis*, H2Aub is associated with a less accessible chromatin state at transcriptional regulatory hotspots, possibly indicating that this less accessible chromatin is poised for activation [29].

PRC1 enzymatic activity is indispensable in mammals, whereas, in *Drosophila*, non-catalytic mechanisms may compensate for partial loss of H2Aub. In plants, H2Aub is critical for development, but redundancy among PRC1 subunits (e.g., RING1A/B and BMI1A/B/C) may mask the full impact of enzyme activity loss. Further studies are needed to resolve the conserved versus lineage-specific roles of PRC1 catalysis.

## 5. The Impact of PcG Proteins on the 3D Chromatin Structure

Higher eukaryotic cells contain approximately two meters of DNA, which must be packaged into a nucleus that is only roughly 10 microns in diameter. Within this confined nucleus, the genome is organized and ordered [79]. Fluorescence In Situ Hybridization (FISH), Chromosome Conformation Capture (3C), and 3C derivative technologies make it possible to detect genomic interactions. Furthermore, research has revealed different scales of chromatin structure: Chromosome Territories (CTs), A/B compartments, Topologically Associating Domains (TADs), and chromatin loops [80,81,82,83]. In mammals and *Drosophila*, PcG proteins exert influences on the organization of 3D chromatin structures across various scales and also take part in the regulation of gene transcription [84,85,86]. In mammals, PcG proteins play a crucial role in mediating chromatin interactions that influence the 3D chromatin structure in stem cell and embryonic development, cell fate determination, and cancer formation [87,88,89,90,91,92]. Specifically, RING1B and PCGF6 facilitate interactions between promoters and enhancers to promote gene expression. In addition, RING1A and RING1B mediate promoter–promoter contacts within the *Hox* gene network and establish a silent but potentially active spatial network that physically constrains developmental transcription factor genes and their enhancers. When cell fate is determined, these genes are selectively released from this spatial network, resulting in transcriptional activation [93,94,95]. vPRC1 is primarily responsible for the ubiquitination of histone H2A; in comparison, cPRC1 is involved in local chromatin compression and long-distance interactions, which are considered to be crucial mechanisms for restricting DNA accessibility and forming repressive nucleosomes [89,92]. Genes modified by H3K27me3, including *Hox* genes, establish reciprocal inter- and intrachromosomal interaction networks within the nucleus. Deletion of Eed leads to a decrease in the frequency of chromatin interactions within polycomb-targeted regions [87,96]. For example, in mouse embryonic stem cells, PRC2 proteins regulate long-range chromatin interactions, collaborating with other gene regulatory networks to shape the 3D chromatin structure [87].

The results of recent studies have also demonstrated that progress has been made in understanding the effects of PcG proteins on the 3D chromatin structures in plants [97]. In *Arabidopsis*, Hi-C analyses revealed that the global genome topology is altered in the *lhp1* mutant. In addition, genes located at both ends of the same LHP1-mediated chromatin loop are highly co-regulated [98]. During the lateral root development of *Arabidopsis*, long non-coding RNA Auxin-regulated Promoter Loop (APOLO) binds to LHP1 to disrupt the chromatin loops associated with LHP1, thereby regulating local chromatin conformation. Such a finding also suggests that LHP1 is involved in the formation of chromatin loops [99]. Modulating the level of H3K27me3 could significantly affect 3D chromatin structure: increasing the level of H3K27me3 promotes the formation of new repressive chromatin loops, whereas decreasing the level leads to chromatin reconfiguration and the activation of gene expression. As a result, H3K27me3 plays a pivotal role in co-regulating genes during plant development by influencing the 3D chromatin structure [100]. The identification of chromatin loops associated with H3K27me3 within gene clusters of *Arabidopsis*, rice (*Oryza sativa*), and soybean (*Glycine max*) indicates that these long-distance chromatin loops are conserved across plant species [101]. While TADs are a prominent feature of the mammalian genome and have subsequently been identified in plants with large genomes, the results of studies focusing on *Arabidopsis* have revealed only a limited number of structural domains associated with H3K27me3 [102,103]. The TAD-like Compartment Domain (CD) was subsequently identified in *Arabidopsis*, where PRC1 and PRC2 collaborate to maintain interactions within the CDs, whereas PRC1 also functions independently to prevent the formation of H3K4me3-associated chromatin loops [104]. Despite evidence demonstrating that PcG proteins regulate gene expression by forming chromatin loops, the precise mechanisms by which these loops influence gene transcription and their variations across different cell types and developmental stages remain to be fully elucidated [98,100,101]. EMF1 interacts with the cohesin subunit Sister-chromatid Cohesion protein 3 (SCC3), and both proteins are colocalized at the CD boundary. The absence of either EMF1 or SCC3 leads to a decrease in CD boundary strength. EMF1 potentially functions as a genome modulator, collaborating with cohesin to maintain CD boundary integrity and regulate gene expression [105]. In particular, it is important to study the mechanism by which PcG proteins and other regulatory factors together affect chromatin structure [106].

**Figure 2:**
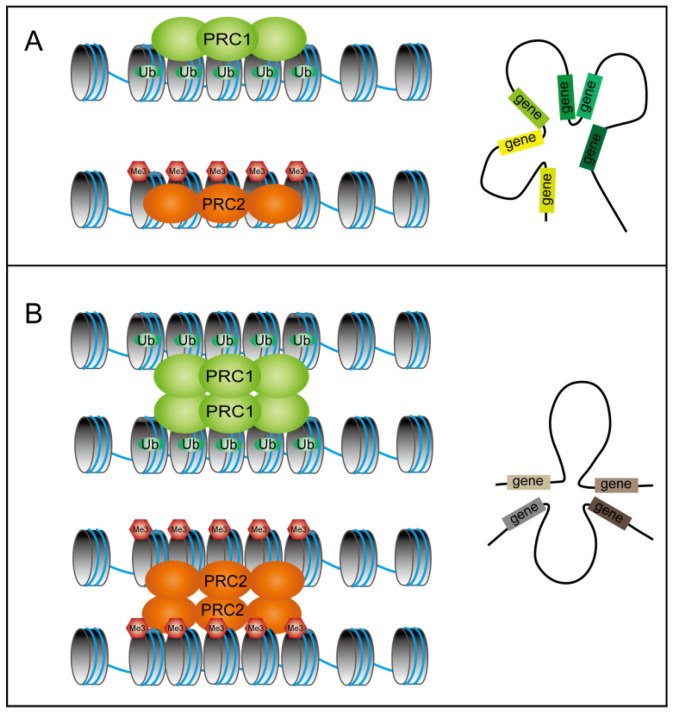
Schematic diagram demonstrating how PRC1 and PRC2 compress chromatin. (**A**) Local interactions of PRC1 and PRC2 may facilitate tight chromatin packing, leading to the synergistic regulation of locally tandem genes. This local interaction ensures coordinated expression or repression of neighboring genes. (**B**) Long-range interactions: PRC1 and PRC2 may also mediate long-range chromatin interactions, enabling the coregulation of functionally related genes that are distantly located in the genome. These interactions help maintain synchronized expression patterns across dispersed genomic regions.

The cPRC1 components PH1/2/3 are crucial for chromatin interactions in mammals, while CBX2 plays a key role in the phase separation of chromatin structure. Additionally, the mammal PRC2 component SUZ12 has the capacity to form homologous dimers [89,92,107,108]. The current research results on the effect of polycomb proteins on 3D chromatin structure in plants indicate that the 3D organization is mainly influenced by histone modifications such as H2Aub and H3K27me3. In the plant PRC1 complex, no homologs of the mammalian PH and CBX proteins have been identified, leading to the ambiguous specific protein subunits responsible for mediating chromatin interactions. However, plant polycomb-mediated chromatin interaction regulation may rely on analogous principles. In mammals, PH and CBX proteins coordinate in local and long-range chromatin contacts through interactions [89,92]. Similarly, studies in *Arabidopsis* have revealed that the BMI1s regulate both local and long-range chromatin interactions [104]. Building on these observations, a conceptual model for plant PRC1/PRC2 chromatin contacts was proposed. This model posits two modes of interaction: (1) local chromatin compaction (Figure 2A), where PRC subunits directly crosslink adjacent nucleosomes, and (2) long-range chromatin interactions (Figure 2B), where PRCs mediate interactions between distant genomic loci. Genes anchored by these loops are regulated by the long-range chromatin interactions.

## 6. The Role of PcG Proteins in the Response to Environmental Stresses

**Figure 3:**
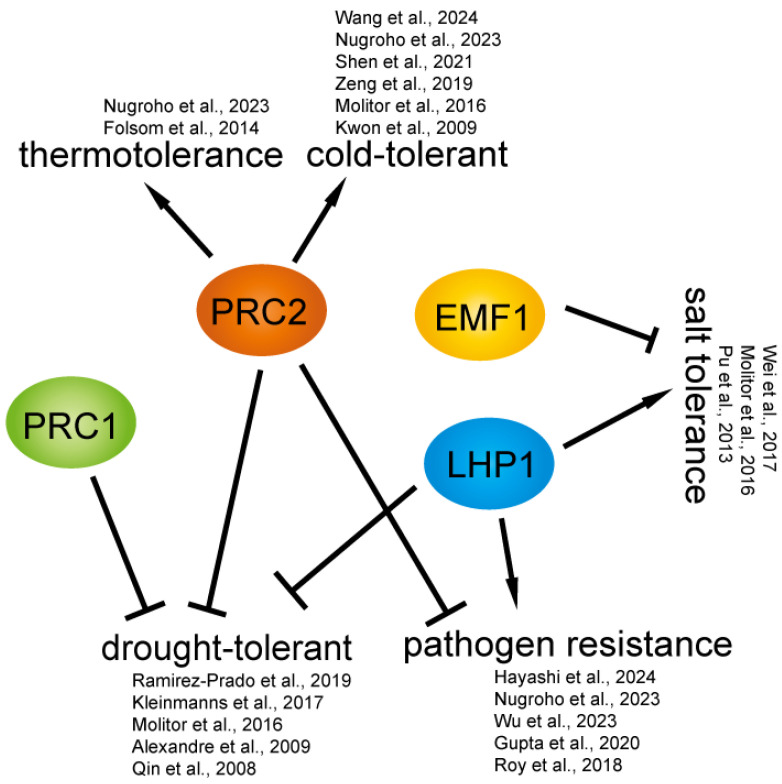
The role of PcG in environmental stress responses. When external stress is present, PcG promptly regulates relevant genes to cope with it. This process has positive implications for plant stress resistance [23,112,113,114,115,116,117,118,119,120,121,122,123,124,125,126,127].

Throughout their life cycle, plants are exposed to a variety of environmental stimuli and have evolved complex mechanisms to sense external signals and respond appropriately. Their responses to various adversities rely heavily on the ability to rapidly and specifically regulate their transcriptome [109]. Environmental factors such as drought, high salinity, high temperature, and cold are detrimental to plants and elicit a range of physiological, biochemical, and molecular responses, significantly impacting crop yields and posing a threat to agricultural productivity [110]. PcG components play a crucial role in responding to environmental stress. Together with Trithorax Group (TrxG) proteins, they contribute to both the immediate response and the establishment of stress memory in plants. Following exposure to abiotic or biotic stresses, plants develop stress memories, enabling them to respond more rapidly and efficiently to subsequent challenges [111]. In plants, PRC1, LHP1, EMF1, and PRC2 can regulate the expression of stress-responsive genes in response to environmental stresses. This process mainly introduces the role of PRC1, LHP1, EMF1, and PRC2 in environmental stresses (Figure 3).

Research shows that BMI1A/B, the components of the PRC1 complex, appear to act as negative regulators in response to drought stress. In *Arabidopsis*, BMI1A and BMI1B have been identified to ubiquitinate Dehydration-responsive Element Binding protein 2A (DREB2A), leading to its degradation via the 26S proteasome. Notably, the *bmi1a/b* mutant increases tolerance to drought stress, indicating that these proteins play a negative role in the drought stress response [23].

LHP1 is involved in drought, salt, cold, and biotic stresses. In *Arabidopsis*, it has been found that the ribonucleoprotein LHP1-interacting Factor 2 (LIF2) interacts with LHP1 and co-targets multiple genes that are primarily involved in stress responses, such as those related to drought, salt stress, and low temperature [112]. *Arabidopsis* LHP1 negatively regulates the MYC2-dependent immune pathway by suppressing the expression of *ANAC019* and *ANAC055*, in addition to their downstream genes. Notably, the *lhp1* mutant displayed heightened sensitivity to Abscisic Acid (ABA) and increased drought tolerance [113]. When wheat (*Triticum aestivum*) is infected by stripe rust, the repression by LHP1 is lifted, resulting in the upregulation of related resistance genes, thereby enhancing disease resistance [114]. The soybean protein GmPHD6, although lacking intrinsic transcriptional regulatory capacity, recognizes hypomethylated histone H3K4me0/1/2 and forms a complex with the transcriptional activators LHP1-1 and LHP1-2. This interaction enables GmPHD6 to activate the expression of downstream genes, ultimately enhancing salt tolerance in soybeans [115].

EMF1 participates in the negative regulation of salt stress tolerance. In *Arabidopsis*, EMF1 is a plant-specific protein involved in the PcG-mediated repression of gene transcription; in comparison, Ultrapetala1 (ULT1) is a TrxG factor that counteracts the action of PcG. Both EMF1 and ULT1 regulate gene expression by modulating histone modifications on target genes. Notably, the deletion of EMF1 enhances salt tolerance in *Arabidopsis*, whereas the deletion of ULT1 diminishes this tolerance [116].

PRC2 responds to drought, salt, cold, thermal, and biotic stresses. Plants regulate interstitial cellular fluid water levels to maintain normal physiological functions, whereas pathogenic bacteria enhance infection by inducing the accumulation of this water. Research has shown that the *Arabidopsis* PRC2 histone methyltransferase subunit CLF plays a critical role in modulating leaf cell interstitial fluid water levels via epigenetic regulation of the ABA signaling pathway and stomatal movement [117]. *Arabidopsis* Blister (BLI) protein serves as a critical regulator of stress-responsive genes through its interaction with PRC2. Mutation of *BLI* results in the upregulation of numerous stress-responsive genes and decreased tolerance to drought stress. By repressing ABA-responsive PRC2 target genes, BLI enhances plant resistance to both cold and drought stresses [118]. In *Arabidopsis,* decreasing the level of *MSI1* led to the upregulation of multiple stress-response-related genes, including those involved in osmotic and salt stress. Additionally, MSI1 can directly bind to the chromatin of the drought-inducible gene *RD20*, indicating that MSI1 plays a negative regulatory role in the drought stress response [119]. The *Arabidopsis mea* mutant demonstrated enhanced resistance to bacterial pathogens indicating that MEA functions as a negative regulator to prevent excessive activation of the immune response. This role helps balance plant growth and defense mechanisms [120]. In *Arabidopsis*, Long-chain Base Kinase 1 (LCBK1) interacts with the PRC2 complex component MEA and enhances stomatal immunity by Phosphorylating phytosphingosine (PHS), thereby boosting plant resistance to bacterial pathogens [121]. BrCLF plays a role in stress signaling and stress-responsive metabolism in *Brassica rapa*, particularly in aliphatic and indolic glucosinolate metabolism. An epigenomic analysis has shown that H3K27me3 is significantly enriched in genes linked to these developmental and stress-responsive processes [122]. Heat stress reduces the duration of the syncytial period during rice seed development, resulting in premature cellularization and, ultimately, a decrease in seed size. The rice PRC2 gene *OsFIE1* shows temperature sensitivity, with its expression levels inversely related to the length of the syncytial stage. These findings indicate that OsFIE1 likely plays a critical role in regulating seed size in response to heat stress [123]. H3K27me3 modulates gene expression and enhances plant (rice, potato (*Solanum tuberosum*), and *Arabidopsis*) adaptation under cold stress conditions [124,125,126,127].

The results of these studies suggest the potential application of PcG proteins in improving crop stress resistance. Unraveling the mechanisms by which PcG proteins modulate stress responses holds promise for developing innovative strategies to enhance crop resilience. Further research is required to investigate how genetic engineering techniques can be employed to regulate the expression and function of PcG proteins, ultimately leading to improved stress tolerance in crops.

## 7. Conclusions

PcG proteins play an important role in regulating the 3D chromatin structure in plants. PcG proteins can mediate chromatin interactions such as PH and CBX proteins in mammals [89,92]. In *Arabidopsis*, RING1s can interact with BMI1s, and PRC1 can recruit PRC2 [7,50]. In previous studies, mutation in PcG catalytic subunits (*CLF*/*SWN*, *MEA*, and *BMI1A*/*B*/*C*) resulted in severe developmental phenotypes in *Arabidopsis* [29,64], which indicate that the enzymatic activities of PcG proteins are the basis to influence on 3D chromatin structure in plants [101,104]. The regulation of 3D chromatin structure by PcG can modulate changes in gene expression [100]. Additionally, PcG proteins play a critical role in *Arabidopsis*, wheat, soybean, *Brassica rapa*, rice, and potato in the responses to high-temperature, drought, low-temperature, and pathogenic stresses. These stress responses may be induced by changes in the 3D chromatin structure regulated by PcG and other regulatory mechanisms, which impact the expression of stress response genes, showing a correlation with plant stress tolerance. The results of these studies can provide a reference for investigating crop stress and improving crop yield.

Taken together, this review summarizes the roles of PcG proteins in modulating 3D chromatin structures in plants and provides examples of their involvement in stress resistance processes. However, the molecular mechanisms underlying the collaborative regulation of the chromatin architecture by PRC1, PRC2 and also the other histone modifications—remain to be further elucidated. Furthermore, due to the incomplete identification of PcG homologous proteins in crops and the technical challenges in genetic validation (e.g., generating stable mutants or transgenic lines), the conserved or species-specific regulatory mechanisms by which PcG complexes interact with diverse stress-response pathways across different crops remain unclear.

## 8. Prospects

Although PRC1 and PRC2 play crucial roles in plant development, their recruitment mechanisms may differ across various cell types and different developmental stages. Moreover, compared to PRC2, it is crucial to determine whether PRC1’s function is contingent upon H2Aub, identify its reader, and ascertain whether PRC1 ubiquitinates other H2A variants beyond H2A and H2AZ. Remarkable progress has been made in studies on the 3D chromatin structure. Despite differences between plant and animal genomes, similar features such as loops, TADs, and A/B compartments have been identified in plants. It is recognized that H3K27me3 is involved in maintaining many 3D chromatin structures. Therefore, it is believed that PcG exerts its functions by regulating these 3D chromatin structures. However, further investigation is required to elucidate how these chromatin structures precisely influence gene transcription and how they vary across different cell types and developmental stages. In particular, understanding the mechanisms through which PcG proteins and other regulatory factors affect the formation and stability of these loops remains a critical area of research. It remains unclear whether certain polycomb proteins in plants can form homologous dimers to mediate chromatin interactions—as they do in mammals—and which polycomb components mediate chromatin interactions. The results of other studies also show that PcG proteins play an important role in stress response. Further in-depth research is required to explore how PcG proteins impact plant growth, development, and adaptation processes and how 3D chromatin structure may be associated with this process. Specifically, it is important to delve into how PcG proteins affect 3D chromatin structure at various developmental stages and under different stress conditions, further influencing gene expression and plant phenotypes. These insights regarding polycomb proteins may pave the way for innovative strategies and approaches to increase agricultural productivity. In-depth research into how PcG proteins regulate the 3D chromatin structure and their roles in plant stress resistance will provide epigenetic insights for agricultural production, offering both theoretical significance and practical value.

## Figures and Tables

**Table 1:** PcG components in plants, mammals, and flies.

PcG	Components	Plants	Mammals	Flies
PRC1	core subunits	RING1A/B	RING1A/B	dRing
		BMI1A/B/C	PCGF2/4(cPRC1) PCGF1/3/5/6(vPRC1)	Psc Su(z)2
	cPRC1 specific	/	CBX2/4/6/7/8	Pc
			PH1/2/3	Ph
			SCM	Scm
	vPRC1 specific	VAL1/2 AL1/2/3/4/5/6/7 NDX VRN1 HDAC SAP18	RYBP/YAF2 KDM2	dRybp dKdm2
PRC2	core subunits	CLF SWN MEA	EZH1/2	E(z)
		EMF2 VRN2 FIS2	SUZ12	Su(z)12
		FIE	EED	Esc
		MSI1	RBBP4/7	Nurf55
Plant specific PcG	subunits	LHP1 EMF1	/	

## Data Availability

No new data were created or analyzed in this study.
